# Determination of Time and Concentration Conditions Affecting Polylactic Acid (Pla) Production

**DOI:** 10.3390/polym17152009

**Published:** 2025-07-23

**Authors:** Jorge Braulio Amaya, Gabriela Vaca

**Affiliations:** Biotechnology Engineering, Universidad Politecnica Salesiana, Cuenca 170517, Ecuador; gvacaa@est.ups.edu.ec

**Keywords:** polylactic acid, polymerization, lactic acid, ring-opening polymerization (ROP), biopolymer

## Abstract

Polylactic acid (PLA) is a renewable biopolymer that has attracted considerable interest due to its ability to replace petroleum-based synthetic polymers, thereby offering a more sustainable alternative to global environmental concerns. This study focused on evaluating the effect of catalyst concentration and reaction time on the efficiency of PLA synthesis via the Ring-Opening Polymerization (ROP) technique. The process involved a lactic acid esterification stage (using 88% lactic acid) to obtain lactide, employing 40% and 60% (*v*/*v*) sulfuric acid concentrations, followed by polymerization at various reaction times (10, 15, 20, and 30 min). Analysis of variance (ANOVA) results revealed that the 40% catalyst concentration had a statistically significant effect on polymer yield (*p* = 0.032), whereas reaction time showed no statistical significance (*p* = 0.196), although the highest yields were recorded at 10 and 15 min. Fourier Transform Infrared Spectroscopy (FTIR) confirmed the presence of the characteristic functional groups of PLA, and Differential Scanning Calorimetry (DSC) revealed a semi-crystalline structure with a high melting temperature, indicating good thermal stability. These results validate the viability of PLA as a functional and sustainable biopolymer.

## 1. Introduction

In recent decades, concerns about the environmental impact of petroleum-based plastics have led to an active search for alternative materials that are sustainable, biodegradable, and environmentally friendly. In this context, PLA has proven to be one of the most promising biopolymers due to its biodegradability, biocompatibility, and excellent mechanical and barrier properties [1]. These characteristics have led to its application in various industries, such as food packaging, medicine, and agriculture [2,3,4,5,6,7].

**Figure 1:**
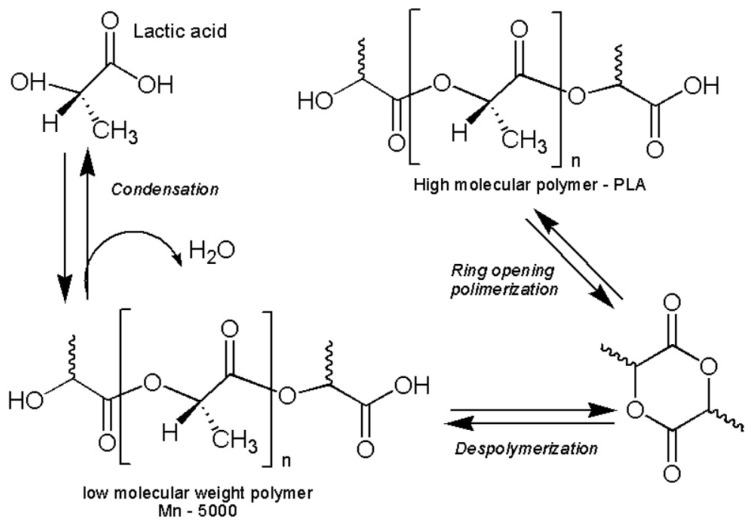
Ring opening polymerization mechanism of PLA from lactide monomer. Source: Adapted from [12].

PLA is an aliphatic polyester obtained by ROP of the lactide monomer (Figure 1), which comes from lactic acid generated by the fermentation of renewable resources such as corn or sugarcane. Its structure imparts exceptional transparency, adjustable molecular weight, recyclability, and compostability [2,7]. These characteristics make it a viable alternative to conventional plastics such as polypropylene, polystyrene, and polyethylene terephthalate [8,9,10,11].

Despite its many advantages, the efficient synthesis of PLA remains a challenge, especially when seeking to maximize its quality and reduce production costs. The nature of the catalysts, reaction time, temperature, pressure, and concentration of the reagents are determining factors in obtaining a polymer with the desired properties. Previous research has shown that the control of these variables can significantly impact the molecular weight and stability of the final product [13,14,15,16]. For example [17,18,19] was able to synthesize PLA with a molecular weight of 30,000 g/mol by optimizing time and catalyst concentration, while [20,21,22] concluded that an appropriate combination of pressure, temperature, and catalyst type significantly improves the polymer properties.

However, these studies have been developed mainly in contexts where there are greater technological resources, which leaves a gap in terms of the adaptability of these processes in developing countries such as Ecuador. Despite having renewable raw materials and regulatory frameworks that promote the circular economy [23,24,25], the problem of plastic pollution remains critical. In 2022, Ecuador produced about 490 million tons of plastic products and a continuous increase is projected [5,25,26]. In light of this situation, the development of bioplastics such as PLA represents a key strategy to reduce dependence on non-degradable materials [23,27].

Furthermore, recent research has revealed that it is possible to modify PLA properties by incorporating additives such as lignin, which can improve its degradation rate [28,29] or its thermal stability [9,30], although they can also negatively affect other characteristics, such as crystallinity. These observations open new questions on the optimization of PLA synthesis for specific applications, based on a balance between performance, cost, and sustainability [31,32,33,34,35].

Several methods have been developed for the synthesis of PLA, including direct polycondensation, azeotropic polycondensation, and ring-opening polymerization (ROP). While direct polycondensation is economically viable, it is limited by low molecular weights due to water retention. Azeotropic techniques improve yields by removing water using solvents, but these methods are less environmentally friendly. ROP, in contrast, enables the production of high-molecular-weight PLA under milder conditions, with better control over polymer structure and properties. In this study, the ROP method was selected to evaluate the influence of reaction time and catalyst concentration on PLA synthesis, using an environmentally sustainable approach suitable for potential application in local contexts such as Ecuador [36].

In Ecuador, there is growing interest in replacing petroleum-based plastics due to increasing environmental regulations, limited industrial recycling infrastructure, and the availability of agricultural residues that could serve as potential feedstock for biopolymers. However, local production of PLA remains limited due to the high cost of lactide and technical barriers to implementing traditional polymerization routes. This research seeks to provide evidence for the efficiency of the PLA production process under conditions adapted to the capabilities of the national environment, and thus contribute to the progress towards a more sustainable economy.

## 2. Materials and Methods

The experimental design incorporated a total of 24 individual samples, each prepared in triplicate to ensure reproducibility. These samples were systematically coded from M1 to M24, where ‘M’ denotes ‘mixture’ or ‘sample’. Samples M1 through M12 correspond to reactions conducted with a 40% (*v*/*v*) sulfuric acid catalyst, while samples M13 through M24 were synthesized using a 60% (*v*/*v*) sulfuric acid catalyst. Within each catalyst concentration group, four distinct reaction durations were investigated: 10, 15, 20, and 30 min.

**Figure 2:**
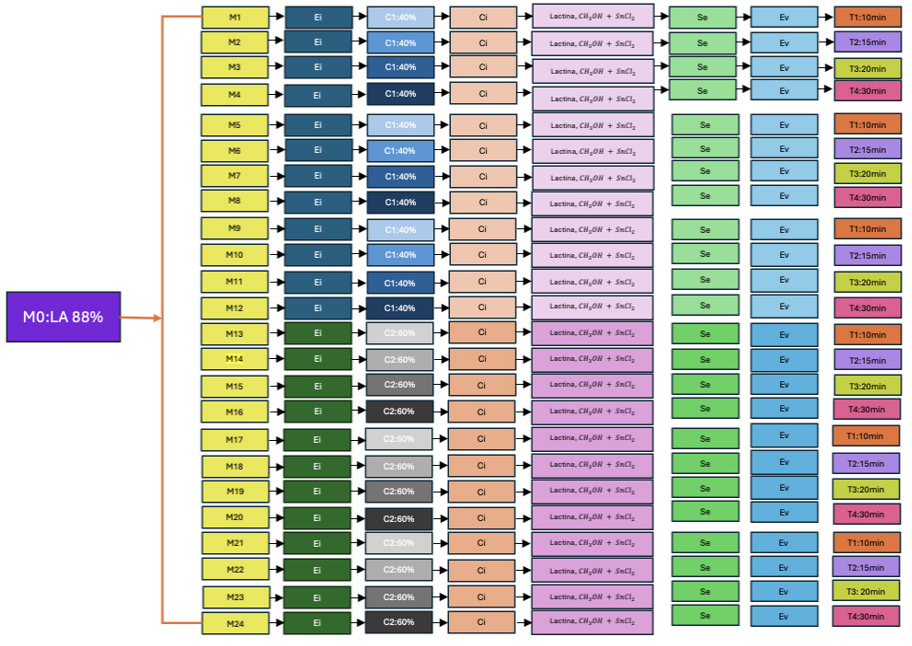
This figure illustrates the experimental design for the synthesis of PLA. Each stage, from esterification to sedimentation and evaporation, is systematically coded to represent the sequence of operations and the experimental variables (acid concentration and reaction time). M0: Lactic Acid 88%; Ei: Esterification; C1: Concentration 1: 40% *v*/*v*; C2: Concentration 2: 60% *v*/*v*; Ci: Heating; Se: Sedimentation; Ev: Evaporation; T1: Time 1: 10 min; T2: Time 2: 15 min; T3: Time 3: 20 min; T4: Time 4: 30 min. Source: The authors.

Figure 2 provides a detailed schematic of this experimental procedure, outlining the specific conditions applied to each sample.

The process began with 88% lactic acid (M0), followed by esterification (Ei), then heating (Ci), sedimentation (Se), and evaporation (Ev). Two sulfuric acid concentrations (C1: 40% *v*/*v* and C2: 60% *v*/*v*) and four reaction times (10, 15, 20, and 30 min) were tested to evaluate their influence on PLA synthesis yield.

### 2.1. Synthesis of PLA from Lactic Acid by ROP

**Figure 3:**
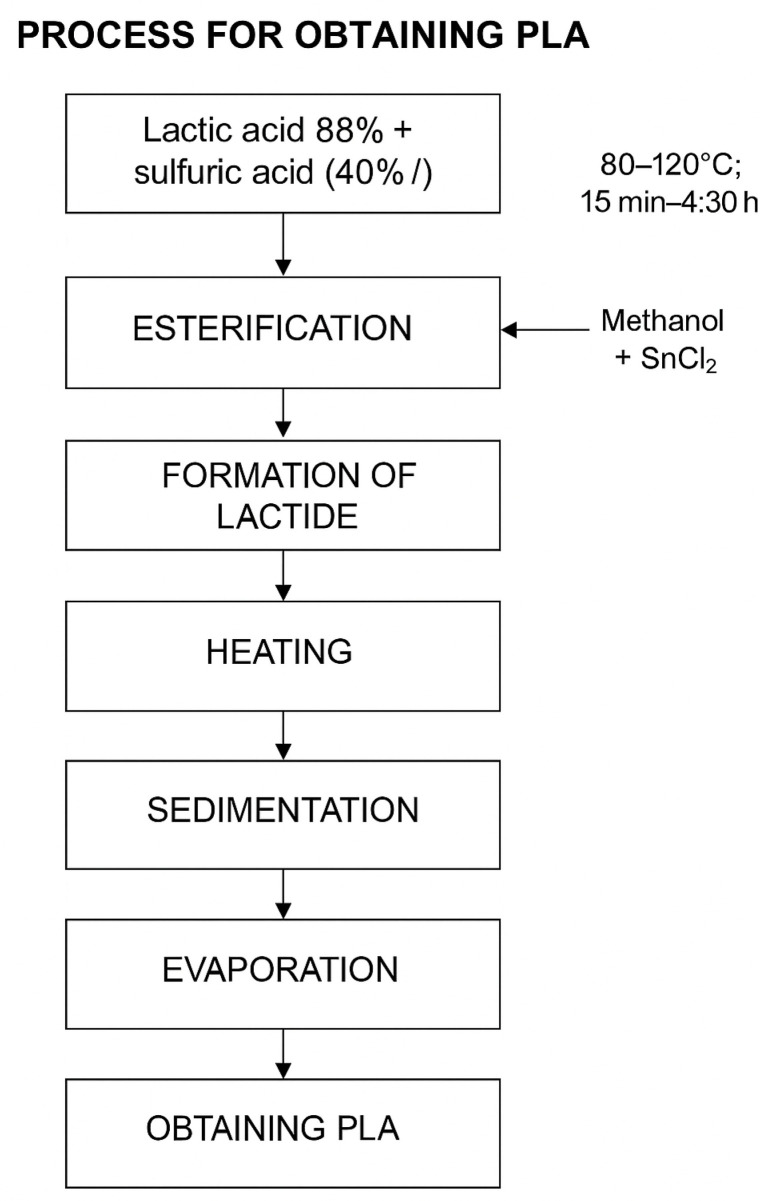
PLA obtaining process. Source: The authors.

The synthesis of PLA from lactic acid initially involves a polycondensation process, whereby water molecules are removed to form linear oligomers. Subsequently, these oligomers can be transformed into lactide, a cyclic dimer of lactic acid, which is used in ROP to obtain high-molecular-weight PLA [8]. In this method, lactic acid first undergoes catalyzed esterification, where the elimination of water favors the formation of ester bonds, a key step for obtaining lactide [12,37]. In the experimental stage, lactic acid (88%) was subjected to a polycondensation process in the presence of sulfuric acid as catalyst. Two concentrations of the catalyst were evaluated: 40% and 60% *v*/*v*. For each experimental condition, three replicates were performed, resulting in a total of 24 samples. In each test, 50 mL of lactic acid was mixed with 2.5 mL of sulfuric acid (according to the corresponding concentration), carrying out the reaction in a thermoagitator plate for 15 min. The dehydration process was carried out by continuous application of heat at a temperature of 120 °C, during periods of 30 min, 3 h and 30 min, and 4 h and 30 min, according to established experimental conditions. At this stage, 2.5 mL of sulfuric acid was added as catalyst, with the objective of favoring the elimination of water generated during the reaction [12,31,38]. This step is essential to promote efficient polycondensation, an indispensable condition for the subsequent formation of the lactide in an adequate manner. This procedure is shown in Figure 3.

#### Heating Process

This stage is crucial, since the heat treatment allows the effective activation of the catalyst, which facilitates the opening of the lactide ring and, with it, the formation of the polymeric chains characteristic of PLA [37]. In this phase, 5 g of lactide was added, followed by the addition of 50 mL of reagent grade methanol (GR) and 3 g of stannous (II) chloride, used as a catalyst. The mixture was subjected to a constant temperature of 60 °C for different time intervals (10, 15, 20, and 30 min). This procedure was repeated in three replicates for each of the concentrations evaluated (40% and 60%), to facilitate the understanding of the procedure.

The selection of catalyst concentrations of 40% and 60% *v*/*v* was based on preliminary experimental observations and relevant literature reports [31,39]. These indicate that concentrations below 40% are often insufficient to promote efficient esterification and lactide formation. This can lead to incomplete polymerisation. Conversely, concentrations exceeding 60% may accelerate side reactions or cause the degradation of intermediates due to excessive acidity and thermal stress. The two chosen concentrations represent a balanced range that allows the influence of catalytic strength on the ROP process to be evaluated, while ensuring the integrity of the monomer and the structural quality of the resulting PLA. This approach also considers practical issues relating to catalyst handling and process safety in laboratory-scale synthesis.

### 2.2. PLA Synthesis Procedure

Polylactic acid (PLA) was synthesized via acid-catalyzed ring-opening polymerization. A mixture of 50 mL of lactic acid (88%) and 2.5 mL of sulfuric acid (40% or 60% *v*/*v*) was stirred magnetically in a 100 mL borosilicate beaker at 60 °C. Four reaction times were tested: 10, 15, 20, and 30 min. After heating, the mixture was subjected to sedimentation and evaporation as detailed in the following sections. No purification steps were applied. Each condition was performed in triplicate.

### 2.3. Sedimentation Process

The mixture was left to stand for a period of 10 min, which allowed, by sedimentation, the PLA particles to agglomerate at the bottom of the flask [40]. This step is essential for the separation of the solid polymeric fraction from the supernatant.

### 2.4. Evaporation Process

In order to eliminate the residual methanol, the recovered product was subjected to a heat treatment in an oven at a constant temperature of 70 °C for one hour. As a result, PLA was obtained in the form of a white powder, a physical characteristic that evidences its solid and dehydrated state. Finally, the material was reserved under controlled conditions for further analysis and characterization.

### 2.5. Final Yield Calculation of PLA

To determine the percentage yield of PLA obtained, Equation (1) was applied:

(1) Yield(%)=((MassofPLAobtained(g)/Initialmassoflacticacid(g))×100

And to calculate the initial mass of initial lactic acid, Equation (2) was used.

(2) Massoflacticacid(g)=Volume(mL)×Concentration(g/mL)

In this study, the following experimental data were considered:

- Volume of lactic acid: 50 mL.
- Lactic acid concentration: 88% (equivalent to 0.88 g/mL).

Therefore, the mass of lactic acid used in each sample was 44 g, calculated as Mass = 50 mL × 0.88 g/mL = 44 g.

Finally, to calculate the average yield of the three best samples corresponding to catalyst concentrations 40% and 60%, Equation (3) was used:

(3) Mean(%)=(∑Individualyields/Totalnumberofsamples)×100

This set of Equations (2) and (3) allowed us to quantitatively evaluate the efficiency of the PLA synthesis process under different experimental conditions.

### 2.6. PLA Identification

For quantitative chemical analysis, an FTIR spectrophotometer available in the Chromatography laboratory, belonging to the “Life Sciences” laboratories, was used. This technique allows characterizing the compounds both at chemical and structural level, in a non-invasive way. On the other hand, the physical characterization was carried out using the DSC method [41,42], which provides detailed information about the thermal properties of PLA.

### 2.7. DSC of the Obtained PLA

The analysis (DSC) was performed using samples of approximately 5 mg. The samples were placed in hermetically sealed aluminum cells, and the analysis was carried out under an inert atmosphere of dry nitrogen, with a constant flow rate of 50 mL/min to avoid oxidation processes during the assay. The thermal protocol employed consisted of three successive stages: *First heating*: Samples were heated from 30 °C to 250 °C at a heating rate of 10 °C/min. The purpose of this stage was to eliminate the thermal history of the polymer, ensuring standardized conditions for subsequent thermal characterization. *Cooling*: After reaching 250 °C, the samples were cooled again to 30 °C, at a controlled rate of −10 °C/min, allowing a homogeneous recrystallization of the material. *Second heating*: Finally, the samples were subjected to a second heating cycle from 30 °C to 250 °C, maintaining the same heating rate of 10 °C/min, in order to determine the thermal transition temperatures of the material. The glass transition temperatures (Tg) were identified at the midpoint of the specific heat increment, while the melting temperatures (Tm) were determined from the maximum of the endothermic peak corresponding to the PLA melting process [20,36,39,41].

## 3. Results

### 3.1. Analysis of the Results of the Yields Obtained from the PLA

Under experimental conditions and applying Equation (1), it was found that at a concentration of 40% sulfuric acid as catalyst, the sample that presented the highest recovered mass of PLA was sample 11 (M11), which, after a reaction time of 20 min, produced 0.65 g of PLA, representing a yield of 1.47%. The second highest recovery was obtained in sample 5 (M5), with 0.56 g of PLA and a yield of 1.27%, with a reaction time of 10 min. Finally, Table 1 shows that the third sample with the highest product mass was sample 10 (M10), which achieved a recovery of 0.45 g PLA, corresponding to a yield of 1.02%, under a reaction time of 15 min. In all cases, the independent variable controlled was the catalyst concentration (40%), while different time intervals were evaluated as experimental variable. These values are detailed in Table 1.

Under the experimental conditions corresponding to a concentration of 60% sulfuric acid, the sample that presented the highest mass of PLA recovered was sample 14 (M14), obtaining 0.45 g and a yield of 1.02%. The second sample with the highest mass was sample 16 (M16), which achieved a recovery of 0.44 g of PLA, corresponding to a yield of 1.00%, under a reaction time of 30 min. Finally, the third sample with the highest recovery was sample 20 (M20), with 0.33 g of PLA and a yield of 0.75%.

### 3.2. ANOVA Analysis

The analysis of variance (ANOVA) performed to evaluate the effect of reaction time, sulfuric acid concentration, and their interaction on the yield in obtaining PLA reveals the following results, as seen in Table 2. The concentration variable showed a statistically significant effect on the yield, with a value of *p* = 0.032 (<0.05), and a value of F = 5.314, indicating that there are significant differences in the yields depending on the concentrations evaluated (40% and 60%). In contrast, the reaction time variable did not present a significant effect (*p* = 0.196; F = 1.786), as did the time–concentration interaction, which was also statistically non-significant (*p* = 0.175; F = 1.976). These results suggest that, within the experimental conditions studied, the catalyst concentration is the factor with the greatest influence on the amount of PLA obtained, while the reaction time and its interaction with the concentration do not generate a statistically relevant effect on the yield of the process.

In the study developed by [37], it is pointed out that the variables concentration and time are determining factors in the process of obtaining PLA. When contrasting the results obtained in that research with those of the present one, it is observed that, although the catalyst concentration has a statistically significant effect on the PLA yield, the reaction time does not present a relevant influence. This discrepancy can be attributed to several experimental variables, among them, the specific operating conditions of each study, such as the differences in the reaction times used for lactide formation, which could have directly affected the efficiency of the polymerization process in both investigations.

**Figure 4:**
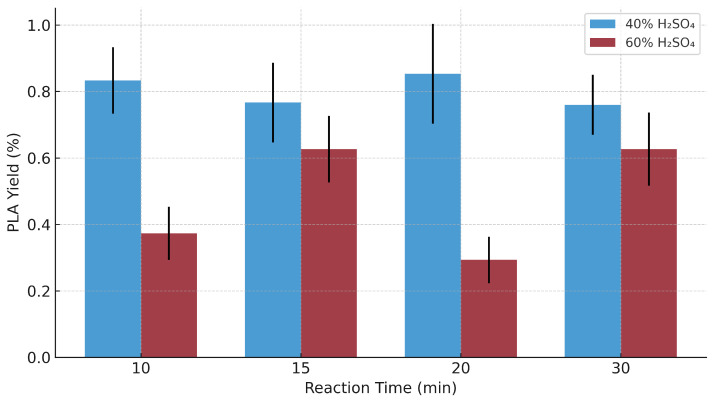
Average PLA yield (%) at different reaction times and catalyst concentrations (40% and 60% *v*/*v*). Error bars represent standard deviation over three replicates per condition. Data have been normalized to percentage yield from initial lactic acid mass.

Figure 4 illustrates the average mass of PLA obtained as a function of reaction time; it can be observed that, in general terms, the 40% concentration tends to generate higher masses of PLA compared to that of 60%, especially at 10, 15, and 20 min. The highest average mass is recorded with the 40% concentration at 10 min, which could indicate that a short time favors polymerization under this concentration. In contrast, at 20 min, a greater variability in the results is evident, particularly for the 40% concentration, suggesting a lower reproducibility under these conditions. Overall, the graph supports the hypothesis that catalyst concentration is a determinant factor in PLA yield, while reaction time, over the range evaluated, does not exert a consistent or significant effect.

The experimental data indicate a clear interaction between sulfuric acid concentration and reaction time in determining PLA yield. Under a constant reaction temperature of 60 °C, the use of 40% *v*/*v* H_2_SO_4_ consistently resulted in higher PLA recovery compared to 60% *v*/*v*, across all time intervals. This outcome suggests that while sulfuric acid acts as a catalyst for ring-opening polymerization and dehydration reactions, higher concentrations (e.g., 60%) may lead to excessive acid-catalyzed hydrolysis or side reactions that limit the accumulation of high-molecular-weight chains, thus reducing the mass of recoverable polymer.

Furthermore, maximum yields were observed at intermediate reaction times (10–20 min), particularly under 40% acid concentration. Extending the reaction time beyond this range did not improve yields and, in some cases, reduced them (e.g., M12 and M24). This trend is consistent with thermal or acid-induced degradation of oligomeric intermediates, which is known to occur when reaction systems are overexposed to acidic environments. These findings confirm that both catalyst concentration and residence time must be finely controlled to avoid kinetic or thermal constraints that impair PLA productivity.

### 3.3. Qualitative Analysis of PLA

#### FTIR Spectrum and Analysis DSC of PLA Obtained

The FTIR spectrum obtained for PLA samples synthesized under 60% concentration conditions and different reaction times (10, 15, 20 and 30 min) evidences the presence of the characteristic bands associated with the functional groups characteristic of polylactic acid, which confirms the formation of this biopolymer.

**Figure 5:**
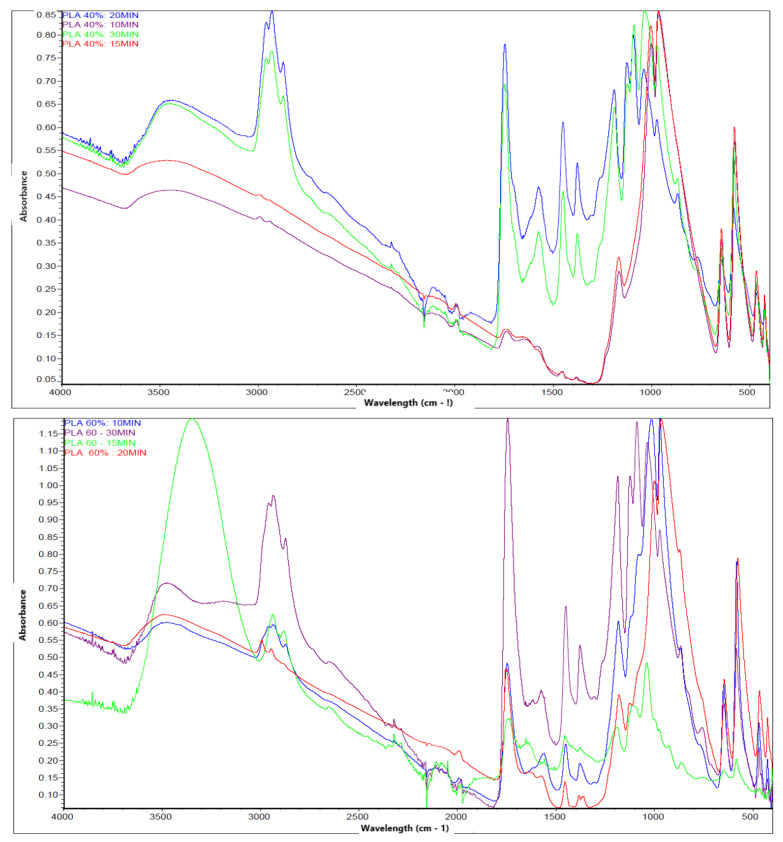
FTIR spectrum of PLA. The upper image belongs to the PLA synthesized with 40% acid and the lower image belongs to the PLA synthesized with 60% acid.

Figure 5 shows that in the 3000–2800 cm^−1^ region, a set of bands attributed to the stretching vibrations of the methyl (-CH_3_) and methylene (-CH_2_-) group, commonly present in the main chain of PLA, is observed. These bands are indicative of the aliphatic structure of the polymer. The presence of an intense and well-defined band in the 1750–1735 cm^−1^ region confirms the stretching vibration of the carbonyl (C=O) group of the ester, which is a distinctive sign of the PLA structure. This band is clearly identifiable in all samples, which supports the effective formation of the polymer. Signals are also identified in the 1180–1080 cm^−1^ region, corresponding to the asymmetric and symmetric stretching vibrations of the C-O-C bond, characteristic of the ester group. These bands, together with those observed around 1450 cm^−1^ (bending of the -CH_3_ group), consolidate the spectroscopic evidence of the presence of PLA in the analyzed samples. The reproducibility of the spectra over different reaction times and replicates indicates that, despite variations in experimental conditions, the chemical structure of PLA is conserved. No extraneous signals or bands attributable to impurities or unwanted intermediates are observed, suggesting an adequate synthesis of the polymer under the established parameters.

In Figure 5 (upper) (40% H_2_SO_4_), the spectra of all samples show strong absorption bands around 1745–1750 cm^−1^, corresponding to the C=O stretching of the ester group, which is a key marker for PLA. Additionally, peaks between 1180 and 1080 cm^−1^ are attributed to C–O–C stretching, and the 2945–2995 cm^−1^ bands are assigned to –CH_3_ and –CH_2_ stretching. The spectra differ slightly in intensity across reaction times (10–30 min), indicating small variations in polymer chain organization or residual unreacted material.

In contrast, Figure 5 (lower) (60% H_2_SO_4_) exhibits broader and more intense absorption bands, especially in the 3500–3200 cm^−1^ region, which may suggest a higher presence of hydroxyl (–OH) groups, likely from incomplete esterification or absorbed moisture. The C=O and C–O–C regions remain visible but with more variability in intensity and peak sharpness. This could reflect either a less uniform polymer structure or more degradation due to the higher acidity and potential side reactions over extended reaction times.

**Figure 6:**
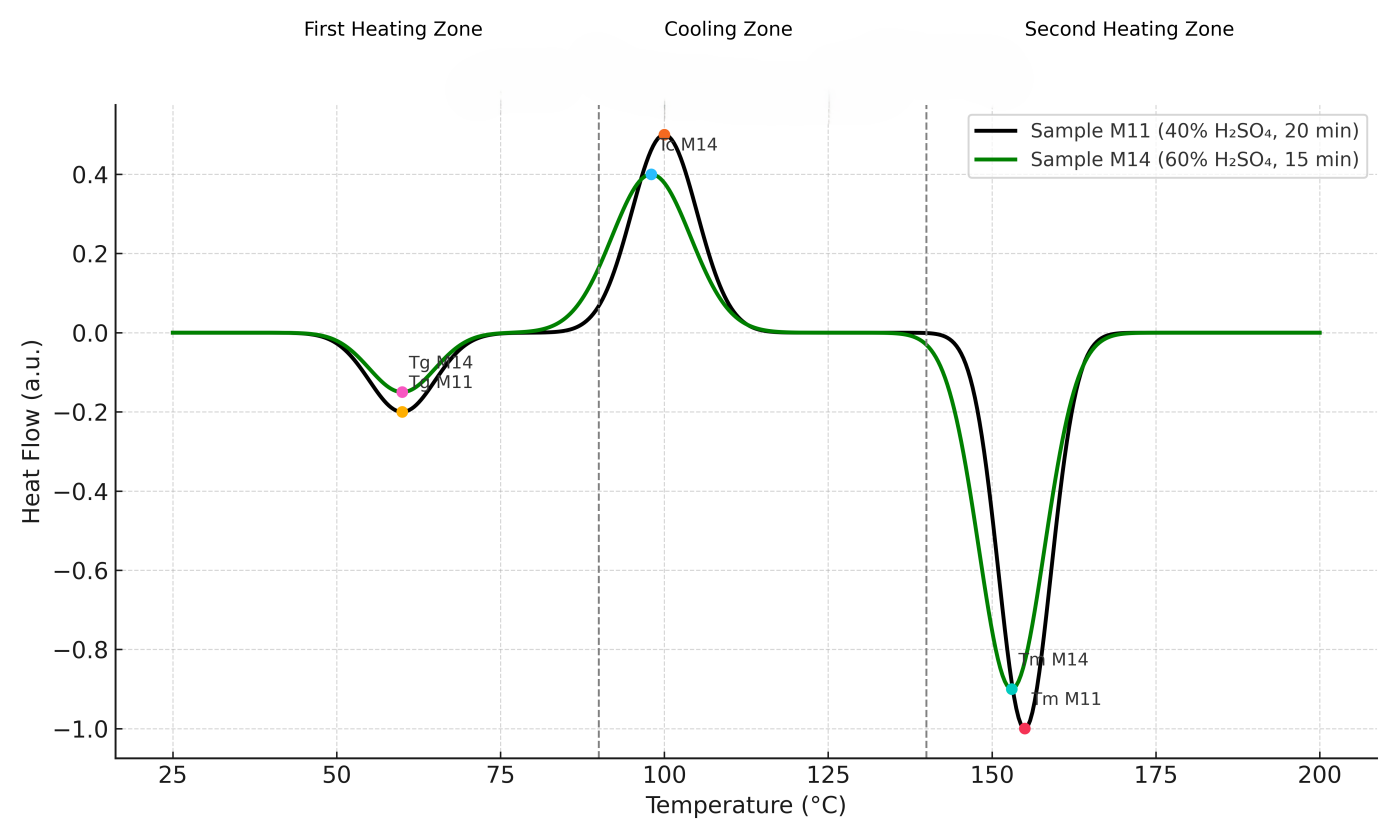
DSC thermograms of PLA samples synthesized under different reaction times and catalyst concentrations. The curves show the thermal transitions of the glass transition temperature (Tg), cold crystallization (Tc), and melting point (Tm).

Regarding the DSC of PLA under different experimental conditions of concentration (40% and 60%) and reaction time (10, 20 and 30 min), Figure 6 shows the following observations: *Glass Transition Temperature (Tg)*: A characteristic drop near 60 °C is observed, which is consistent with the thermal behavior of PLA, indicating the onset of molecular mobility in the amorphous phase. *Cold Crystallization Temperature (Tc)*: The treatment at a lower concentration and time presents an exothermic peak around 100 °C, signaling the structural reorganization of the polymer. *Melting Temperature (Tm)*: Endothermic peaks are detected between 152 °C and 155 °C, corresponding to the melting of the PLA crystalline phase. The “60%-30 min” condition presented a higher melting point, which could be related to a higher crystallinity induced by the thermal and catalytic treatment. This analysis suggests that both catalyst concentration and reaction time affect the thermal structure of the synthesized PLA, evidenced by shifts in Tg, Tc, and Tm.

## 4. Discussion

The experimental results demonstrate a significant influence of sulfuric acid concentration on the yield of polylactic acid (PLA) synthesized via ring-opening polymerization. Analysis of variance (ANOVA) revealed that the catalyst concentration exhibited a statistically significant effect on the final PLA mass, evidenced by a *p*-value of 0.032 (Table 2), indicating a notable difference in the obtained polymer yields when comparing the 40% and 60% catalyst concentrations. Conversely, the reaction time (ranging from 10 to 30 min) did not exert a statistically significant effect on the polymer yield, with a *p*-value of 0.196. This observation suggests that, within the investigated temporal parameters, reaction duration is not a primary determinant for enhancing PLA production efficiency in this synthesis protocol.

The literature supports the importance of controlling catalyst concentration during PLA synthesis. Refs. [37,43,44] also identified concentration as a critical variable in polymer yield; however, in their study, reaction time had a significant impact. This discrepancy could be attributed to differences in experimental conditions, such as the synthesis method used or the stage of lactide obtaining, which can modify the overall kinetics of the process.

From a quantitative approach, the 40% concentration allowed a higher average yield (1.25%) to be achieved compared to the 60% concentration (0.923%). Among the most notable samples are M11 (1.47%), M5 (1.27%), and M10 (1.02%). The trend observed in the bar chart with confidence intervals (Figure 4) reinforces this observation, showing a clear superiority in yields under the 40% condition, especially in short reaction times (10 and 15 min). This difference could be explained by more efficient formation of reactive intermediates and less degradation of intermediate products, a phenomenon that usually occurs at excessively high acid concentrations, as pointed out by [30], who demonstrated statistically significant effects of catalyst concentration and reaction time on PLA yield. Although the final yields were below 1.5%, these results represent an initial step toward optimizing low-cost production methods for PLA in small-scale or resource-limited settings.

FTIR spectroscopic analysis confirmed the presence of the main functional groups characteristic of PLA, such as carbonyl (C=O), methyl (CH_3_), ether (C–O), and hydroxyl (OH), in accordance with the chemical structure reported by [30,45,46]. It is important to note that, under the condition of 40% concentration, the absorption bands were sharper and more intense, suggesting greater purity or better structural definition of the synthesized polymer.

The DSC curve of PLA shows a glass transition temperature (Tg) close to 60 °C, a crystallization peak around 100 °C during cooling, and endothermic melting between 152 and 155 °C during the second heating. These thermal events are characteristic of the semi-crystalline behavior of PLA and are consistent with values reported in previous studies by [36,47,48], where Tg varies between 55 and 65 °C and Tm between 150 and 160 °C, depending on the degree of crystallinity and the thermal conditions applied. These results confirm the ability of PLA to reorganize its structure under controlled thermal cycles.

Taken together, the experimental results allow us to establish a clear correlation between the acid concentration and the structural and thermal quality of the PLA obtained. Although the reaction time did not show a statistically significant effect, the thermal and structural behavior of the polymer seems to benefit from moderate times and controlled concentrations. These findings are fundamental for optimizing the PLA synthesis process in future research.

One limitation of this study is the absence of molecular weight distribution data and elemental composition analysis, which are essential for a more comprehensive characterization of the synthesized PLA. Future work will focus on determining the molecular weight using gel permeation chromatography (GPC) and verifying chemical purity through CHNS/O elemental analysis, in order to better correlate structural features with thermal and mechanical behavior.

## 5. Conclusions

The highest PLA yield obtained under the current experimental setup was 1.47%, corresponding to sample M11. While this yield is relatively low, it reflects the outcome of a simplified ring-opening synthesis without product purification, and under initial screening conditions. The ANOVA statistical analysis revealed that the concentration of sulfuric acid used during polymerization is the only variable with a significant effect on obtaining polylactic acid (PLA), with a value of Pr(>F) = 0.032, the 40% concentration being the most efficient by reaching an average yield of 1.23%, compared to 0.92% recorded with 60%. In contrast, the heating time variable (10, 15, 20, and 30 min) did not present significant influence (Pr(>F) = 0.196), although the data suggest that times of 10 and 15 min could slightly favor PLA production. Similarly, the interaction between time and concentration did not show statistical significance (Pr(>F) = 0.175), ruling out a synergistic effect between both conditions. On the other hand, structural analysis by FTIR confirmed the presence of functional groups characteristic of PLA in all conditions. Samples synthesized with 40% sulfuric acid showed more uniform spectral profiles, while those obtained with 60% also presented the key functional groups, albeit with slightly greater intensity variation. These differences do not indicate structural deficiencies, but rather reflect the sensitivity of the reaction to acid concentration and time. Differential scanning calorimetry (DSC) allowed identifying a glass transition temperature (Tg) between 60–65 °C, a melting temperature (Tm) between 152 and 155 °C, and a cold crystallization temperature (Tc) of 130.23 °C with a crystallization enthalpy of 28.83 J/g, indicating that the material obtained has a semi-crystalline nature and good thermal stability.

Based on the experimental findings, we recommend using a 40% sulfuric acid concentration with short reaction times (10–15 min) to maximize PLA yield while maintaining good thermal and structural properties. These conditions are cost-effective and compatible with moderate technological infrastructures, making them suitable for implementation in decentralized or small-scale production settings in developing countries.

Compared to other studies employing sulfuric acid or similar catalysts for PLA synthesis, the yields obtained in this work are relatively low; however, they are consistent with early-stage syntheses that do not include purification steps or solvent removal [19]. The glass transition and melting temperatures observed (60 °C and 155 °C, respectively) fall within the expected ranges reported in the literature [4,20,47], confirming that the resulting material exhibits typical thermal behavior of PLA. The simplified methodology explored here provides an accessible alternative for small-scale production and educational applications, particularly in regions where access to advanced infrastructure or high-purity lactide is limited [4].

## Figures and Tables

**Table 1:** Experimental conditions and final PLA yield obtained under different combinations of reaction time and sulfuric acid concentration. All experiments were conducted using 50 mL of lactic acid (88%) and 2.5 mL of sulfuric acid, under constant stirring at 60 °C. Recovered mass corresponds to dry PLA after evaporation.

Sample	Time (min)	Catalyst (% *v*/*v*)	Volume (mL)	Temp. (°C)	PLA (g)	Yield (%)
M1	10	40	2.5	60	0.32	0.72
M2	15	40	2.5	60	0.32	0.72
M3	20	40	2.5	60	0.36	0.81
M4	30	40	2.5	60	0.43	0.97
M5	10	40	2.5	60	0.56	1.27
M6	15	40	2.5	60	0.38	0.72
M7	20	40	2.5	60	0.27	0.61
M8	30	40	2.5	60	0.38	0.86
M9	10	40	2.5	60	0.37	0.84
M10	15	40	2.5	60	0.45	1.02
M11	20	40	2.5	60	0.65	1.47
M12	30	40	2.5	60	0.33	0.75
M13	10	60	2.5	60	0.22	0.50
M14	15	60	2.5	60	0.45	1.02
M15	20	60	2.5	60	0.25	0.56
M16	30	60	2.5	60	0.44	1.00
M17	10	60	2.5	60	0.27	0.61
M18	15	60	2.5	60	0.28	0.63
M19	20	60	2.5	60	0.11	0.25
M20	30	60	2.5	60	0.33	0.75
M21	10	60	2.5	60	0.07	0.15
M22	15	60	2.5	60	0.21	0.47
M23	20	60	2.5	60	0.08	0.18
M24	30	60	2.5	60	0.17	0.38

**Table 2:** Analysis of variance (ANOVA).

	DF	Sum Sq	Mean Sq	F Value	Pr (>F)
Time	1	0.0350	0.03497	1.786	0.196
Concentration	1	0.1040	0.10402	5.314	0.032 *
Time–Concentration	1	0.0387	0.03868	1.976	0.175
Residuals	20	0.3915	0.01958		

* *p* < 0.05 indicating a statistically significant result. This suggests that concentration has a significant effect on the dependent variable (mass) under the conditions of the experiment).

## Data Availability

The original contributions presented in this study are included in the article. Further inquiries can be directed to the corresponding author.

## Abbreviations

PLA	Polylactic Acid
DSC	Differential Scanning Calorimetry
ANOVA	Analysis of Variance
Tg	Glass Transition Temperature
Tm	Melting Temperature
FTIR	Fourier Transform Infrared Spectroscopy
GR	Reagent Grade Methanol
ROP	Ring-Opening Polymerization
